# Tissue is the issue and tissue competition. Re-biopsy for mutation T790: where and why?

**DOI:** 10.1186/s40169-017-0135-8

**Published:** 2017-01-18

**Authors:** Paul Zarogoulidis, Mina Gaga, Haidong Huang, Kaid Darwiche, Aggeliki Rapti, Wolfgang Hohenforst-Schmidt

**Affiliations:** 1 Pulmonary Department-Oncology Unit, ``G. Papanikolaou`` General Hospital, Aristotle University of Thessaloniki, Thessaloniki, Greece; 2 7th Respiratory Medicine Department and Asthma Center, Athens Chest Hospital ‘Sotiria’, Athens, Greece; 3 Department of Respiratory and Critical Care Medicine, Changhai Hospital/First Affiliated Hospital of the Secondary Military Medical University, Shanghai, China; 4 Department of Interventional Pneumology, Ruhrlandklinik, University Hospital Essen, University of Essen-Duisburg, Essen, Germany; 5 Second Pulmonary Clinic, ‘Sotiria’ Chest Diseases Hospital, 11527 Athens, Greece; 6 Medical Clinic I, “Fuerth” Hospital, University of Erlangen, Fuerth, Germany

**Keywords:** Lung cancer, Egfr, t790, Biopsy, Ebus

## Abstract

Lung cancer is still the leading cause of death among all cancers. During the last 15 years, pharmacogenomics of lung cancer have established targeted therapy with tyrosine kinase inhibitors (TKIs) for epidermal growth factor receptor (EGFR) positive patients in adenocarcinoma or mixed adenosquamus lung cancer patients. However; while novel drugs are released in the market, at the same time novel mutations are observed after tyrosine kinase inhibitor administration. Recently the novel mutation T790 was observed and is highly prevalent in patients already treated with a TKI. A new drug targeting this mutation is already on the market, however; the most important factor for successful treatment in these patients, is adequate tissue re-sampling so that novel mutations can be detected.

## Background

Lung cancer is still diagnosed at a late stage disease, mainly due to the lack of early disease symptoms and the absence of an effective screening strategy. Therefore most patients are diagnosed at a non-operable stage and systematic treatment has to be administered. Nowadays, non-small cell lung cancer (NSCLC) treatment includes non-specific cytotoxic agents in the form of chemotherapy and anti-vascular endothelial growth factors. Moreover, in the last 15 years, pharmacogenomics as well as focused research in adenocarcinoma resulted in targeted treatments for epidermal growth factor receptor (EGFR) and anaplastic lymphoma kinase positive patients (ALK). These two groups of patients are currently candidates for oral kinase inhibitors in the form of a capsule. Nowadays we have already have 1st, 2nd and 3rd generation TKIs as well as ALK inhibitors in the market [1–3]. Right now ROS1 pathway can be inhibited with crizotinib. Moreover; immunotherapy has just been established for NSCLC, although several drugs were already on the market for other malignancies such as melanoma [4].

## Main Text

In order to diagnose and molecularly characterize lung cancer, adequate tissue sampling is paramount and several techniques are being used such as; bronchoscopy, endobronchial ultrasound (radial–linear), biopsy under CT guidance, or even surgery. In everyday practice, most of these procedures are performed by interventional pulmonary physicians, while radiologists and thoracic surgeons are also involved in tissue sampling when required. Current biopsy techniques acquire cytology samples and tissue biopsies, or even cytological sample with tissue fragments. The sample depends on the biopsy equipment and the technique that the physician is using. Most centers and physicians use the equipment that they have available with the technique that is safer for the patient. The sample is then forwarded to the cytology or pathology laboratory for diagnosis. However; we should keep in mind that for several biopsy techniques more advanced sample diagnostic procedures such as cell blocks have to be utilized [5]. So, one of the issues that have to be resolved before any biopsy procedure, is the proper handling of the acquired sample. After confirmation of diagnosis, both cytology or histology samples can be used for molecular testing in order to investigate EGFR, ALK, KRAS, ROS1, BRAF and MET mutations. Molecular testing is usually performed for EGFR and ALK mutations but other markers such as KRAS, ROS1, BRAF and MET mutations can be tested and, within clinical studies, medications are available [6–8]. However, these other treatments have not been approved for use in lung cancer. And, in all cases, enough tissue should be sampled so that the diagnosis is accurate and molecular testing can be performed. Although, there are diagnostic platforms who can investigate EGFR mutations using just 3 cells, this does not allow the investigation of other molecular pathway over-expressions [9]. Currently molecular biologists along with pharmaceutical companies have established liquid biopsy techniques [10]. Although; these techniques still have a lower yield they could be used in those patients where re-biopsy is not possible due to performance status [11]. It must be taken into account that a positive liquid biopsy result is considered acceptable for diagnosis, however, a negative result does not rule out positivity of the lung cancer tumor cells or at the site of metastases.

So tissue is the issue: research into lung cancer treatment is booming and is leading to the rapid development of new medications. As more targeted treatment options become available, testing for multiple markers is required and abundant, good quality samples need to be acquired [12]. Moreover, we have come to understand that lung cancer cells mutate and change throughout treatment and therefore the molecular characterization of these tissue samples acquired both at diagnosis and at relapse is pivotal in guiding treatment decisions second line treatment and beyond second line.

## Discussion

As an example of the research and evolution in lung cancer, within 10 years of the first TKI use in clinical practice, a new mutation appeared in far higher proportions in treated patients, T790, a mutation that makes them non-responsive to first and second generation TKIs [13]. A novel TKI was developed and is already on the market, ossimertinib, AZD9291 [14]. It is therefore imperative to re-examine and re-biopsy the patients with disease relapse under anti-EGFR-TKI [15]. Major issues for these patients are where to perform the re-biopsy and whether the patient is fit for re-biopsy. In cases where the patient is not fit for re-biopsy, liquid biopsy is an option. In the study by Oxnard et al. [16] data presented that, upon availability of validated plasma T790M assays, some patients could avoid a tumor biopsy for T790M genotyping. As a result of the 30% false-negative rate of plasma genotyping, those with T790M-negative plasma results still need a tumor biopsy to determine presence or absence of T790M. Moreover; this not invasive method could be used during the follow up of the patient mutation status. However, where re-biopsy is possible, current clinical practice indicates that re-biopsy has to be performed at the site where relapse was observed, whether the primary site, a lymph node or a distant metastasis such as in the liver and, where the necessary equipment or experience is not adequate then the patient has to be referred to an experienced tertiary hospital. Performing re-biopsy at the site of relapse increases the rate of acquiring tissue harboring the novel mutation T790 and allows the patient a new chance for treatment. Finally, it should be stressed that fresh samples are always preferable in order to perform molecular testing. Tissue samples kept even under the best conditions in paraffin blocks, will still degrade the sample microenvironment so new biopsies are always advisable for the molecular characterization of the lung cancer in cases of relapse and before any changes in treatment decisions. Therefore probably re-biopsy should be proposed when necessary to the patient.

## Conclusion

There has been an effort for non-interventional methods to investigate the status of T790, however; these efforts are still in initial stages [17]. Biopsy samples are still necessary and every effort has to be made towards early diagnosis and targeted treatment.

## Abbreviations

EGFR: epidermal growth factor receptor
ALK: anaplastic lymphoma kinase
KRAS: V-Ki-ras2 Kirsten rat sarcoma viral oncogene homolog
ROS1: Proto-oncogene tyrosine-protein kinase 1
BRAF: proto-oncogene B-Raf
MET: tyrosine-protein kinase Met
TKI: tyrosine kinase inhibitor
NSCLC: non-small cell lung cancer
CT: computed tomography
